# Temporal regularity increases with repertoire complexity in the Australian pied butcherbird's song

**DOI:** 10.1098/rsos.160357

**Published:** 2016-09-14

**Authors:** Eathan Janney, Hollis Taylor, Constance Scharff, David Rothenberg, Lucas C. Parra, Ofer Tchernichovski

**Affiliations:** 1 Department of Psychology, Hunter College, CUNY, New York, NY, USA; 2 Macquarie University, Sydney, Australia; 3 Freie Universität, Berlin; Institute of Biology, Berlin, Germany; 4 Department of Humanities, New Jersey Institute of Technology, Newark, NJ, USA; 5 Department of Biomedical Engineering, City College of New York, CUNY, New York, NY, USA

**Keywords:** birdsong, music, butcherbird, complexity, temporal regularity, aesthetic balance

## Abstract

Music maintains a characteristic balance between repetition and novelty. Here, we report a similar balance in singing performances of free-living Australian pied butcherbirds. Their songs include many phrase types. The more phrase types in a bird's repertoire, the more diverse the singing performance can be. However, without sufficient temporal organization, avian listeners may find diverse singing performances difficult to perceive and memorize. We tested for a correlation between the complexity of song repertoire and the temporal regularity of singing performance. We found that different phrase types often share motifs (notes or stereotyped groups of notes). These shared motifs reappeared in strikingly regular temporal intervals across different phrase types, over hundreds of phrases produced without interruption by each bird. We developed a statistical estimate to quantify the degree to which phrase transition structure is optimized for maximizing the regularity of shared motifs. We found that transition probabilities between phrase types tend to maximize regularity in the repetition of shared motifs, but only in birds of high repertoire complexity. Conversely, in birds of low repertoire complexity, shared motifs were produced with less regularity. The strong correlation between repertoire complexity and motif regularity suggests that birds possess a mechanism that regulates the temporal placement of shared motifs in a manner that takes repertoire complexity into account. We discuss alternative musical, mechanistic and ecological explanations to this effect.

## 1. Background

Many oscine songbirds learn and perform an impressive variety of songs. The European nightingale (*Luscinia megarhynchos*), northern mockingbird (*Mimus polyglottos*) and pied butcherbird (*Cracticus nigrogularis*) have acquired notoriety for their singing virtuosity [1]. Song complexity can be advantageous in mating and in social interaction because it can signify life experience [2], learning capabilities [3], affiliation with a local culture of song dialects [4] and it can prevent habituation in female listeners [5,6]. On the other hand, performing a large variety of song types may increase the difficulty with which conspecific listeners memorize and evaluate them. Thus, it might be easier for birds to produce [7], perceive [8,9] and remember [10] songs if their temporal patterning is structured [11,12].

This trade-off between diversity and regularity has an interesting parallel in human music, which maintains a characteristic balance between repetition and novelty [13–15]. Tuning the levels of predictability can affect a listener's behavioural state: the appeal of repetition is a reflection of our adaptive preference for predictability [16,17], while moderate variability elicits a behavioural state of increased arousal [12]. In most musical compositions, repetition and variation are carefully balanced, avoiding extremes that lead to habituation or overload [18–21]. In this manner, balancing repetition and variation in rhythms, pitch intervals or timbres can affect emotions—creating expectation, anticipation, tension, release or surprise [22,23].

Might similar principles explain some of the structure of vocal performances in songbird species whose singing behaviour and song-syntax composition tend to be complex [24–33]? In the context of aggressive interactions among males, predictability in singing behaviour may signal the level of aggression in territorial dispute [34]. But in the context of an uninterrupted singing performance, balancing variability and predictability might be more complicated: performance diversity may demonstrate virtuosity and prevent habituation, while regularity might assist the listener to recognize patterns. Proper balance could therefore serve to affect the avian listener's behavioural state. If this hypothesis is correct, then the level of temporal regularity in singing behaviour should be balanced against complexity: individual birds with larger song repertoires should aim at higher temporal regularity, and vice versa.

**Figure 1.**
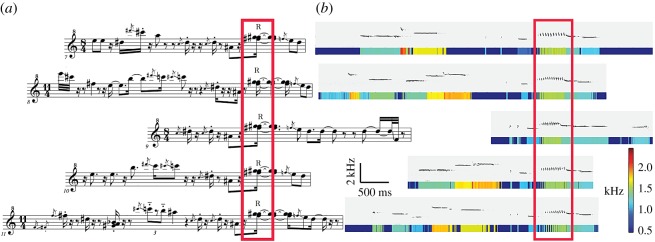
Notation of butcherbird song. (*a*) Musical transcriptions of five butcherbird phrases. An example motif (re-used note, syllable or grouping) is marked by a box. ‘R’ indicates a rattle sound. (*b*) Each phrase from (*a*) is represented as a sonogram and a pitch colour bar (below). The motif boxed in (*a*) is also boxed in (*b*).

**Figure 2.**
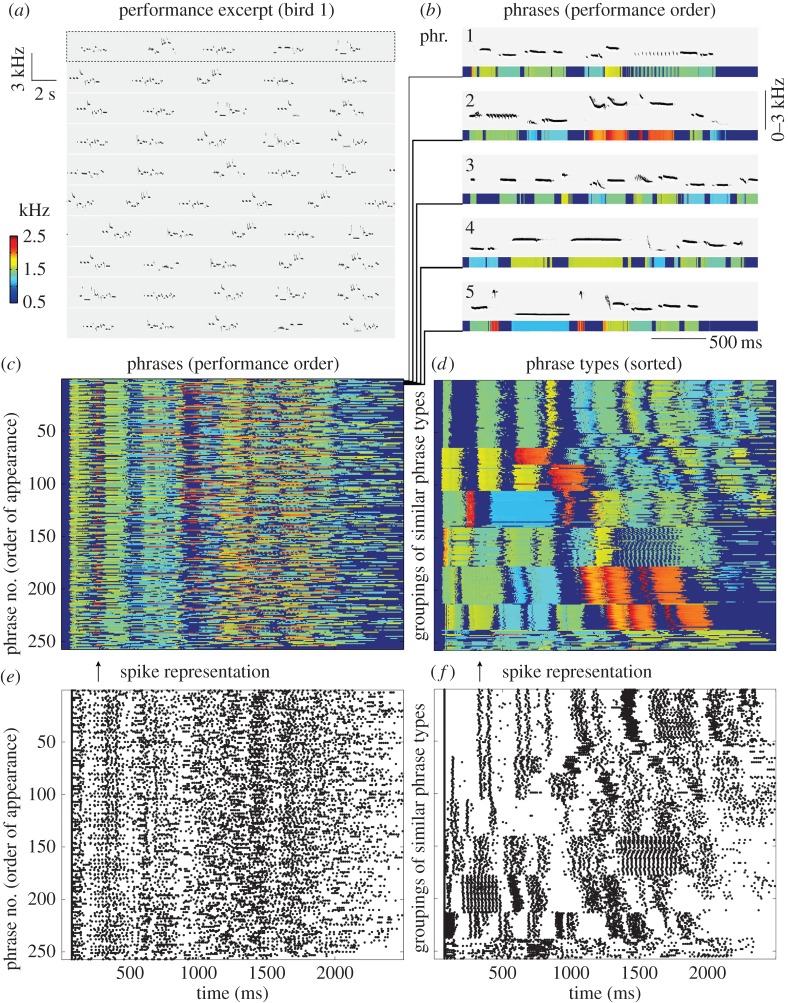
Butcherbird song structure. (*a*) A sonogram of butcherbird song as it occurs naturally, including pauses. (*b*) The first five phrases from *A* (performance order), as sonograms, pictured above false-colour bars representing pitch, and aligned by the onsets of their first syllables. (*c*) About 250 phrases stacked from top to bottom in performance order (see *b* for detail of rows 1–5). Phrase types from (*c*) were (*d*) sorted into types by (*e*) representing them as spike trains (*f*) and using a spike-sorting algorithm to cluster similar patterns.

Here, we investigated this question in free-living Australian pied butcherbirds (*Cracticus nigrogularis*). Their songs are ideal for studying regularity across levels of song structure [1] because song units (notes, phrases) are both complex and easy to identify (figures 1 and 2). Butcherbird vocalizations can be similar in sound to a piping flute, a cornet or an organ [35] and also have inspired composers (such as Olivier Messiaen), who have referred to timbre, contour, gesture, rhythm, repetition, scales and formal structure [36] as meaningful parameters of butcherbird vocalizations. Musicians have also identified overlaps with the human sense of musicality in the butcherbird's development and recombination of melodic motifs [1]. Figure 1 presents a musical transcription of butcherbird phrases alongside sonograms and pitch false-colour bars of the same phrases. A motif common to several unique phrases is highlighted.

The putative melodic motifs in butcherbird song, which musical transcription appears to show, are good candidates to test for balance between repertoire complexity and temporal regularity. To test whether such motifs can be identified through acoustic analysis, for each bird, we recorded and analysed hundreds of uninterrupted song phrases from nocturnal solo performances of identified birds. These performances can span several hours, with a moderate number of phrase types, and strong individual variability in song complexity [36]. Using methods for visualizing and analysing regularity across the hierarchy of song structure over large datasets [37,38], we automatically classified song phrases into types and then detected shared motifs across these phrases. We then examined whether individual birds present their singing repertoire in a manner that balances between repertoire complexity and temporal regularity. We conclude by discussing our results in a broader framework, and present alternative musical, mechanistic and ecological explanations for the balance we observed between repertoire complexity and temporal regularity.

## 2. Results

In order to avoid statistical biases, we randomly divided our data into two sets (cohorts). The first cohort (cohort 1, *n* = 9 birds) was used for data exploration and development of statistical estimates. The second cohort (cohort 2, *n* = 8 birds) was used for testing hypotheses based on the exploration of cohort 1. We first present the exploratory analysis of cohort 1 and then the confirmation of our findings in cohort 2.

### 2.1. Regularity over entire singing performances

Pied butcherbird solo nocturnal song consists of phrases lasting approximately 2.5 ± 0.7 s, typically followed by slightly longer silence intervals [1]. These performances often include hundreds of phrases (figure 2*a*). To examine an entire singing performance, we constructed time course raster plots, where each line represents a phrase and colour indicates the pitch of each syllable (figure 1*b*). Looking at the singing performance of one bird over 25 min (figure 2*c*), we see a fairly homogeneous temporal regularity of repetitive patterns. We first tested if classifying phrases into types can capture this regularity. Distinct phrase types characterized by a stereotyped sequence of notes are often apparent in the pied butcherbird song. However, a more careful inspection reveals a continuum, where some ‘types’ appear to be closely related, while others are not [36]. We therefore implemented an automatic spike-sorting algorithm [39] to automatically classify song phrases into types (figure 2*d*–*f*). We then sorted the raster plots according to those types, and continued sorting and classifying within each type, until all variants of phrase types were grouped (figure 2*d*). The sorted raster plot shows that the temporal regularity is, in fact, a homogeneous ‘mix’ of a rich repertoire of phrase types. We observed a similar effect in all nine birds (electronic supplementary material, figure S1): each time course raster plot appears highly uniform, but the sorted raster plots reveal several phrase types, with high variability in repertoire size across birds.

**Figure 3.**
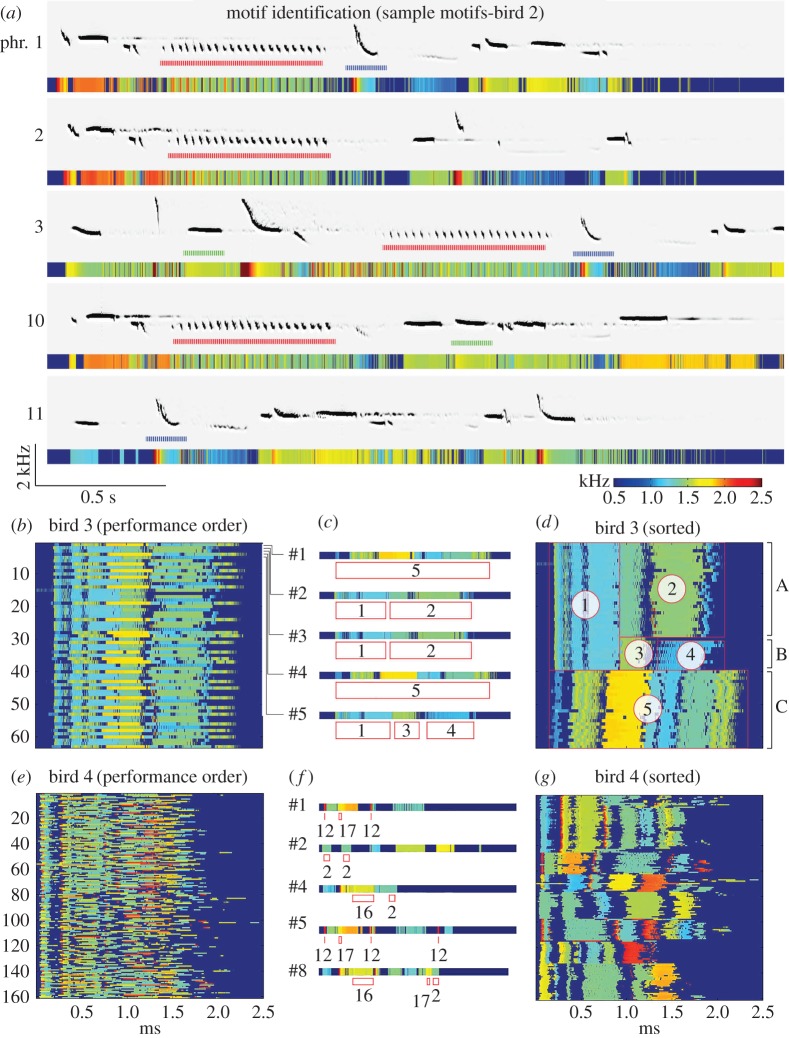
Shared motifs. (*a*) Examples of shared motifs (notes, or groups of notes) that occur across phrase types. Phrase numbers indicate performance order. Motif types are denoted by colours (red, blue and green). (*b–d*) Shared motifs: (*b*) in the order that the bird sang them (top to bottom) and (*d*) according to phrase type. (*c*) Enlargements of the first five phrases from (*b*), highlighting motif reuse. (*e–g*) Same as in *b–d* for a bird with a rich repertoire. Red boxes do not highlight every motif to avoid clutter.

The sorted raster plots reveal why many phrase types appear to be closely related: many phrase types share groups of notes, which we call *shared motifs* (figures 1 and 3). We identified motifs of similar features (duration, pitch and Wiener entropy) across different phrase types. For example, the bird represented in figure 3*b*–*d* had a relatively simple repertoire with only three main phrase types (A, B and C) and five motifs (1–5), where *motif 1* is shared across two phrase types A and B. This bird primarily sings A (1-2), B (1-3-4) and C (5). Shared motifs could be easily identified in the sorted raster plots of all of the birds in our sample (figure 3; electronic supplementary material, figure S2). Therefore, temporal regularity can be judged either at the level of phrases (i.e. if the bird repeats phrase types regularly), at the level of the shared motifs (i.e. if the bird repeats motifs types regularly) or across these levels (i.e. if the temporal order of phrase types is ‘designed’ to regularize the shared motifs).

### 2.2. Statistical regularity in phrase types versus shared motifs

**Figure 4.**
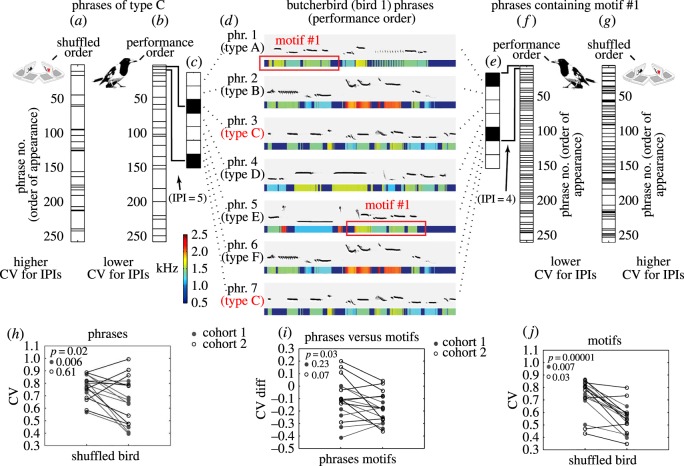
Temporal regularity in phrases versus shared motifs. A binary graph shows reuse of phrase C in an entire performance (approx. 250 phrases) (*a*) when shuffling phrases versus (*b*) using the bird's original phrase ordering. (*c*) A binary analysis of (*d*) the first 7 phrases, marking use of phrase C with filled boxes. The interphrase-interval (IPI) quantifies the number of phrases between occurrences. (*e–g*) Same as in (*a–c*, mirror order) except showing reuse of a motif (highlighted by the red boxes in *d*). (*h*) Across 17 birds shuffled phrases do not achieve the same phrase regularity (measured by ${\mathrm{CV}}_{\mathrm{ph}}^{*}$), however, the effect is weak. (*i*) Motif distribution is more regular than phrase distribution within a performance when compared with within shuffled phrase order. (*j*) The bird's phrase ordering achieves significantly more regular motif distribution than does shuffled phrase order (measured by ${\mathrm{CV}}_{m}^{*}$).

In cohort 1 (*n* = 9 birds), we tested which of the two levels—phrase types or shared motifs—explains more regularity over the entire singing performance of each bird. In order to assess temporal regularity at the level of phrase types, we counted the number of phrases that occurred between two renditions of a phrase type (figure 4*a*–*d*). We refer to this measure at the interval between the recurrences of each phrase type. Similarly, in order to assess temporal regularity at the level of shared motifs, we measured the interval between recurrences of each motif type by counting the number of phrases that occurred between two renditions of a motif (figure 4*d*–*g*). We then calculated the coefficient of variation (CV) of those intervals for each phrase and motif type as estimates of regularity (low CV indicating high regularity and high CV indicating low regularity). Note that in both cases we used the same units (number of phrases in-between) to calculate the CV.

Let us consider, for example, a hypothetical bird that sings four phrase types, A, B, C and D, with a single motif, *i*, shared between phrase types A and B (A^i^ and B^i^), but not in C or D. If the singing performance is completely regular at the phrase level, say ABCD, ABCD…, then the intervals between phrase type renditions all equal (four phrases), and therefore the CV_ph_ = 0. However, the intervals for renditions of the shared motif *i* (A^i^B^i^CD A^i^B^i^CD A^i^…) will alternate between one and three phrases (A^i^B^i^ and B^i^CDA^i^), and therefore CV_m_ = 0.5. Another bird may sing irregularly at the level of phrase types, say ACADBDADBCBD, but nonetheless completely regularly at the level of the shared motif (A^i^ C, A^i^ D, B^i^ D, A^i^ D, B^i^ C, B^i^ D). In this case, CV_ph_ = 0.75 while CV_m_ = 0. To obtain a single (independent) measure for each bird for the temporal regularity in phrase types and motif types, we normalized (equation (5.1)) and pooled (equation (5.2)) across all phrase types (${\mathrm{CV}}_{\mathrm{ph}}^{*}$) and across all motif types (${\mathrm{CV}}_{m}^{*}$) produced by the bird. In sum, ${\mathrm{CV}}_{\mathrm{ph}}^{*}$ and ${\mathrm{CV}}_{m}^{*}$ are scalar estimates which allow us to compare the temporal regularity across phrase and motif types regardless of variance in their relative frequencies within or across birds.

For each bird, we calculated ${\mathrm{CV}}_{\mathrm{ph}}^{*}$ and ${\mathrm{CV}}_{m}^{*}$. We then shuffled phrase order randomly, and recalculated those measures (figure 4*h*,*j*). Comparing observed versus shuffled *measures*, we found that both phrase types (figure 4*h*) and motif types (figure 4*j*) recur more regularly than would be expected by chance. However, the effect was stronger at the level of shared motifs (figure 4*h*–*j*, phrase types: *Mean* ${\mathrm{CV}}_{\mathrm{ph}}^{*}=0.61,$ ${\mathrm{CV}}_{\mathrm{ph}[\mathrm{shuffled}\;\mathrm{data}]}^{*}=0.78,$ *t*_8_ = 3.70, *p* = 0.006; shared motifs: *Mean* ${\mathrm{CV}}_{m}^{*}=0.52,$ ${\mathrm{CV}}_{m[\mathrm{shuffled}\;\mathrm{data}]}^{*}=0.73,$ *t*_8_ = 7.42, *p* = 0.0001). Note also that, in both phrase and motif levels, CV values tend to be higher and less variable in the shuffled data. This may suggest that the temporal order of both phrases and motifs are more regular than expected by chance, but also that a subset of birds in our sample was particularly regular. To further validate this effect, we collected and analysed a new set of data from an additional eight birds. Results confirmed regularity at the shared motifs level (*Mean* ${\mathrm{CV}}_{m}^{*}=0.57,$ ${\mathrm{CV}}_{m[\mathrm{shuffled}\;\mathrm{data}]}^{*}=0.71,$ *t*_7_ = 2.74, *p* = 0.03) but not at the phrase level (*Mean* ${\mathrm{CV}}_{\mathrm{ph}}^{*}=0.73,$ ${\mathrm{CV}}_{\mathrm{ph}[\mathrm{shuffled}\;\mathrm{data}]}^{*}=0.76,$ *t*_7_ = 0.53, *p* = 0.60). Analysis of variance across the two cohorts (electronic supplementary material, table S1) confirmed no significant effect of cohort and no interactions, allowing us to pool the results of those cohorts for further analysis. Across both cohorts motif regularity was significantly higher than phrase regularity (figure 4*i*, *Mean* ${\mathrm{CV}}_{\mathrm{ph}\text{--}\mathrm{ph}[\mathrm{shuffled}\;\mathrm{data}]}^{*}=0.10,$ ${\mathrm{CV}}_{m\text{-}m[\mathrm{shuffled}\;\mathrm{data}]}^{*}=0.18,$ *t*_17_ = 0.245, *p* = 0.03).

### 2.3. Estimating birds' ‘efforts’ to regulate performance of shared motifs

Given that temporal regularity was stronger at the level of shared motifs, we wanted to estimate the extent to which the sequences of phrases produced by a bird facilitate temporal uniformity in shared motifs. In order to answer this question, we need to take the bird's repertoire size into account: the more phrase types and shared motif types a bird can perform, the larger the range of temporal intervals it can produce, and therefore, the ‘potential’ for irregularity in performing shared motifs is higher. We wanted to estimate the level of temporal regularity in performing shared motifs in reference to this potential, which can be judged by the range of possible ${\mathrm{CV}}_{m}^{*}$ values that the bird's singing repertoire can generate. To be conservative, we kept the distribution (histogram) of phrase intervals unchanged, and also kept the structure (and entropy) of transition probabilities between phrase types unaltered. Therefore, focusing on the pairwise transition frequencies between phrase types produced by a bird (i.e. at the level of a first-order Markov model, or bigrams), we inquire as to what extent those pairwise transitions are optimized to increase motif temporal uniformity, that is, to minimize the temporal variability in the production of shared motifs, ${\mathrm{CV}}_{m}^{*}$.

**Figure 5.**
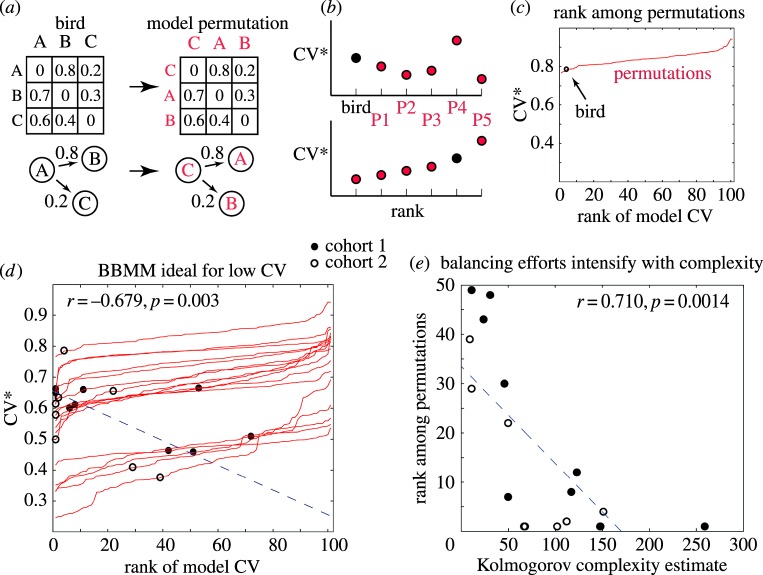
Complexity is counterbalanced by regularity in shared motifs. (*a*) Top-left: simulated bigram transition matrix of phrases; bottom-left: transition diagram between two phrases. Right: permuted transitions matrix and permuted transition diagram. (*b*) For each permuted matrix (P1, P2…), the ${\mathrm{CV}}_{m[\mathrm{synth}\text{-}\mathrm{permuted}]}^{*}$ is calculated (top) and then sorted from lowest to highest (bottom). Sorting situates the rank of the bird's bigram Markov model—${\mathrm{CV}}_{m[\mathrm{synth}\text{-}\mathrm{bird}]}^{*}$, (‘Bird’, black circle)—with respect to the potential range of temporal variability of that bird. (*c*) The process outlined in *B* is applied to one bird—the bird's ${\mathrm{CV}}_{m[\mathrm{synth}\text{-}\mathrm{bird}]}^{*}$—is ranked 4 (out of 100). The ${\mathrm{CV}}_{m[\mathrm{synth}\text{-}\mathrm{bird}]}^{*}$ among the permutations estimates the effort required to produce the low CV*. (*d*) As in *C*, across all birds. Dotted line shows the correlation between the bird's rank and ${\mathrm{CV}}_{m[\mathrm{synth}\text{-}\mathrm{bird}]}^{*}$. (*e*) Birds with higher transitional complexity balance it with increased regularity in motif production.

Figure 5*a* presents a hypothetical example of a bird singing three phrase types, A, B and C, with pairwise transition probability of 80% from *A* → *B* and 20% from *A* → *C*. If we swap the odds (20% for *A* → *B* and 80% for *A* → *C*), ${\mathrm{CV}}_{\mathrm{ph}}^{*}$ must remain unchanged but ${\mathrm{CV}}_{m}^{*}$ may change if those song phrases share different motifs. Therefore, we can construct two pairwise (bigram) Markov models, one for the original and one for the shuffled (permuted) phrase transitions. Running those models, we generated synthetic phrase sequences, and computed synthetic ${\mathrm{CV}}_{m}^{*}$ values for each model. Comparing the means of ${\mathrm{CV}}_{m}^{*}$ values obtained from each model, we can now judge if the original (bird's) phrase transition probabilities are organizing the shared motifs less or more regularly than the alternatives. In sum: we keep the transition probabilities between phrases unaltered except for shuffling the identities of phrase types. These manipulations keep ${\mathrm{CV}}_{\mathrm{ph}}^{*}$ constant but may alter ${\mathrm{CV}}_{m}^{*}$. Each one of these permutations is used to generate synthetic songs, and for calculating a ${\mathrm{CV}}_{m[\mathrm{synth}]}^{*}$ value. Across all the possible permutations, only one corresponds to the phrase transitions produced by the bird, ${\mathrm{CV}}_{m[\mathrm{synth}\text{-}\mathrm{bird}]}^{*},$ which we compare to values obtained from all other permutations ${\mathrm{CV}}_{m[\mathrm{synth}\text{-}\mathrm{permuted}]}^{*}$. With this approach, we next tested to what extent the bird's bigram rules for phrase transitions are ‘optimized’ for increasing regularity at the level of shared motifs.

For each bird, we calculated the bigram transition matrix across all pairs of phrase types. We used that transition matrix to generate synthetic phrase sequences, and compute ${\mathrm{CV}}_{m[\mathrm{synth}\text{-}\mathrm{bird}]}^{*},$ which is our baseline. We then performed 100 permutations of this matrix. For each permutation, we computed ${\mathrm{CV}}_{m[\mathrm{synth}\text{-}\mathrm{permuted}]}^{*}$ (figure 5*b*). We then sorted them and ranked among them the ${\mathrm{CV}}_{m[\mathrm{synth}\text{-}\mathrm{bird}]}^{*}$. Figure 5*c* presents the results for one bird. The potential range for temporal variability in shared motifs can be judged by the range of ${\mathrm{CV}}_{m[\mathrm{synth}\text{-}\mathrm{permuted}]}^{*}$ values, sorted from small to large. The bird's ${\mathrm{CV}}_{m[\mathrm{synth}\text{-}\mathrm{bird}]}^{*}$ is denoted as a black circle. A rank in the neighbourhood of 50 would indicate chance level. Here, the rank is 4 (out of 100), indicating that the birds' phrase syntax brings the temporal regularity of motif recurrence close to its theoretical maximum. Note that the bird's rank order is a direct probability estimate (*p* = 0.04) of how unique the variability of the bird's performance is when compared with similarly complex song, with respect to the shared motifs.

Figure 5*d* presents the results across all 17 birds. Each curve represents the entire range of ${\mathrm{CV}}_{m[\mathrm{synth}\text{-}\mathrm{permuted}]}^{*}$ for one bird. We can see two effects: first, as expected, a bird's phrase transitions produced higher regularity in the temporal order of motifs compared with permutations (mean rank = 17.53, s.d. = 18.30, sign test significantly below the 50th percentile, *p* = 0.00002). This result confirms that each bird's transition probabilities between phrase types are ‘designed’ to achieve much more regularity in shared motifs than the permutations can commonly achieve. Note, however, that our estimate includes only the regularity that can be captured using a first-order Markov model. It captures much, but not all, of the regularity in shared motifs produced by the singing behaviour of the bird (motifs: *Mean* ${\mathrm{CV}}_{\left[\mathrm{synth}\text{-}\mathrm{bird}\right]}^{*}=0.58,$ ${\mathrm{CV}}_{\left[\mathrm{bird}\right]}^{*}=0.54,$ *t*_16_ = 2.44, *p* = 0.027).

Second, across birds we see a strong negative correlation between ${\mathrm{CV}}_{m[\mathrm{synth}\text{-}\mathrm{bird}]}^{*}$ and its rank (*n* = 17, *r* = −0.679 *p* = 0.003). That is, in birds where the range of variation (${\mathrm{CV}}_{m[\mathrm{synth}\text{-}\mathrm{permuted}]}^{*}$) is high, phrase transitions (${\mathrm{CV}}_{m[\mathrm{synth}\text{-}\mathrm{bird}]}^{*}$) are exceedingly selective toward high regularity. Indeed, in the 12 birds where the potential range for variability was high (0.5–0.9), the mean rank was very low, 7.5%, while in the five birds where the potential range for variability was low (0.38–0.48), the mean rank was 41.5%, close to chance level. These results are consistent with the hypothesis that birds take their repertoire complexity into account when adjusting the level of temporal regularity in their singing performance.

In order to test directly for a relationship between complexity and regularity in motif distribution, we tested for correlation between the ${\mathrm{CV}}_{m[\mathrm{synth}\text{-}\mathrm{bird}]}^{*}$ rank, and the Kolmogorov complexity of the bird's bigram transition matrix (figure 5*e*). We used the rank of the ${\mathrm{CV}}_{m[\mathrm{synth}\text{-}\mathrm{bird}]}^{*}$ among the permutations (${\mathrm{CV}}_{m[\mathrm{synth}\text{-}\mathrm{permuted}]}^{*}$) as an approximation of the effort necessary to maintain each bird's motif regularity. We found a strong correlation between transitional complexity and this effort (*r* = 0.710, *p* = 0.0014), confirming that birds with high song complexity tend to produce higher regularity in shared motifs and vice versa.

## 3. Discussion

We explored two levels of temporal regularity in the singing performance of individual pied butcherbirds: at the level of song phrase types, and at the level of shared motifs (stereotyped groups of notes that reappeared across different phrase types). Performances showed high temporal regularity, but most of this regularity was not in the phrase order. It was in the repetition of motifs that are shared across phrase types. The temporal regularity of those shared motifs appear to be balanced against variability: in singing performances of birds with a rich repertoire, phrase syntax was exceedingly optimized (ranked low for CV* among the permutations) to achieve temporal regularity of shared song motifs, whereas in performances from birds with less complex repertoire, temporal regularity was much lower.

Our results are consistent with the hypothesis of an active process, which regulates the temporal diversity of dominant themes in a manner that takes repertoire size into account. However, our results should be interpreted with caution because we could only record a single session from each bird and we might have failed to capture the bird's entire singing repertoire or its performance variability. Further, there are several alternative and complementary explanations to the correlation we observed. Those could involve constraints stemming from song learning and production mechanisms, or feedback from avian listeners, and from constraints on audience perception, memory and engagement. Below we present alternative mechanistic and ecological factors that could potentially explain our results:

1. *Repertoire size and song stereotypy may increase with age:* we do not know the age and sex of the individual birds we recorded. Judged by song stereotypy, and by the context of recording (during breeding season), we suspect that all the birds we recorded from were adults. However, pied butcherbirds appear to be open-ended song learners, and we suspect that, like many other songbird species who learn complex songs, their song repertoire [40] and consistency [41] should increase with age. With respect to temporal regularity of singing behaviour, and how it might change with age, evidence is sparse [3,42]. One may suspect that temporal regularity should increase with development as singing behaviour becomes increasingly more stereotyped. If true, this could explain our findings without any need to consider a mechanism for active balance between repertoire size and regularity. That is, the correlation we observed could be the outcome of two independent effects of song development: the increase in repertoire size and the increase in stereotypy in sequential order. However, as elaborated below, song development may also increase song combinatorial complexity, potentially increasing the potential for irregularity.
2. *Combinatorial complexity may increase with development:* to our knowledge, the effect of age on temporal regularity across song phrases was never studied systematically. But at the syllabic level, studies showed that, with development, combinatorial complexity tends to increase [43]. This effect was demonstrated across two species of songbirds and also in human infants [43]. If a similar increase in combinatorial complexity occurs also at the level of song phrases, than older pied butcherbirds should have both larger repertoires and less constraints on producing complex sequences. Therefore, development may have two opposing effects on motif regularity. An increase in song stereotypy would increase temporal regularity, whereas the increase in combinatorial complexity might decrease it.
3. *Song production memory may improve with development:* mechanisms of song production are relatively well understood, at both acoustic (phonology) and combinatorial levels [43–46]. In Bengalese finches (*Lonchura striata domestica*), for example, transition probabilities are approximately, but not fully Markovian [47,48]. In canaries (*Serinus canaria*), long-range statistical dependencies in syllable order can be detected over several transitions [29]. From a mechanistic viewpoint, such effects indicate long-lasting motor memories. The correlation between repertoire complexity and sequential regularity could reflect developmental changes in motor production memory capacities. If a pied butcherbird cannot hold memory of a shared motif, it would produce it randomly after some time. But if it can, the likelihood of producing that motif again would increase as time passes. As in Konrad Lorenz's ‘action specific energy’ [49], a bird's tendency to produce a behavioural pattern may increase over time, depending on memory capacity. Therefore, a possible explanation for our results is a maturation of the bird's ‘instinctive’ tendency to repeat shared motifs with age. Developmental changes in vocal production memory could therefore account for such an effect.
4. *Feedback from audience, listeners’ attention and memory constraints:* from an ecological perspective, the function of singing behaviour is to alter the behaviour of bird listeners. Feedback from the ‘audience’ could then influence song structure [50]. Several classical studies showed that singing behaviour might be modified in an ‘action-based’ manner [51]. For example, feedback from audience, particularly from females [52], can guide singing behaviour. More recent studies showed that it is easy to train birds to change transition probabilities of song elements using negative reinforcement [47,53]. Assume, for example, that a potential mate prefers singers with large repertoires [54] of accurately performed song phrases [6]. Since an avian listener has limited memory and attention span [55], it can only keep memories of a limited sequence of song elements. Given that a bird needs to listen to several renditions of each motif type in order to assess the accuracy of singing performances, we can draw the following conclusion: in birds whose song repertoire size is small, higher temporal diversity should reduce habituation and facilitate the rate in which bird listeners can sample phrase and motif types. In this case, the small repertoire size is unlikely to exceed the memory limitations of listeners. Therefore, singing with lower temporal regularity should have a positive effect. However, birds with large repertoire size might experience a different reaction from their listeners: when temporal diversity becomes high, listeners may no longer be able to keep track and may not be able to judge singing accuracy, because they cannot hold memory of previous renditions of phrases and motifs. In this case, higher temporal regularity could potentially facilitate memorization and improve song outcome. Therefore, constraints on the memory and attention span of bird listeners can potentially feed back to singing performance, and explain our results.

In sum, at the phenomenological level, the existence of a characteristic balance between repetition and variation is an interesting link between birdsong and music. Regardless of its root cause, the temporal patterns in which singing performance keeps returning to shared motifs can provide a sense of regularity and familiarity for the listener. At the same time, shared motifs return in different contexts (within different phrase types), allowing for a flexible presentation and possibly maintaining a listener's interest.

## 4. Conclusion

To our knowledge, there is no universal correlation between complexity and repetition across musical cultures. Statistical regularities in music can only rarely be collapsed across all musical cultures. For example, some composers (e.g. Philip Glass) are famous for their highly repetitive music. However, balance between repetition and variation is highly abundant in music (within styles) and is one of the most-studied universals in music [16,17,56–60]. Though it is unlikely to apply to all, we have demonstrated in one species of songbird that the overall repertoire complexity of the bird is ‘balanced’ by regularity in temporal structure. To our knowledge, such an effect has not been rigorously documented in non-human animals. We hope that our methods for high-throughput analysis across entire singing performances of wild birds will facilitate studies testing for similar effects in other species.

## 5. Material and methods

### 5.1. Song recordings

The pied butcherbird singing performances were digitally recorded at a sample rate of 44.1 kHz and 16 bits in Australia between 1992 and 2007, using a Sennheiser ME67 shotgun microphone covered with a Rycote windshield, and a minidisc recorder. We restricted our analysis to recordings from 17 birds, where background noise was low and the individual bird could be clearly identified for at least 5 min of continuous singing behaviour. Mean recording length per bird was 32 min, ranging from 5 min to 1 h. Table 1 presents the geographical, seasonal and subspecies distribution of recordings. The two subspecies are not identifiable in the field. *Cracticus nigrogularis nigrogularis* is found in mainland eastern Australia and *Cracticus nigrogularis picatus* in the mainland west. No individual in this study is from their putative contact zone in northwest Queensland. The table therefore presents tentative sub-species identification. We did not find any audible differences in singing behaviour across subspecies, or in any of the measures presented in this study. Breeding takes place primarily in September through November but as early as July and as late as December [61]. All recordings were made principally during the breeding season. Since pied butcherbirds are sedentary, the distances between locations make it very unlikely that the same bird was recorded more than once. Note that each bird was recorded only once and, despite the extended period of recording sessions we might have not managed to sample the full repertoire of each bird, which would have required recording across different singing performances from the same bird over multiple sampling periods. Therefore, the level of comparison made here is not between individual birds *per se*, but between individual recordings taken from individual birds.

**Table 1.** Birds recording data including the location site, recording date and subspecies.

location	day/month/year; time	subspecies	recordist
Great Wall of China near Hall's Creek, Western Australia	03/09/2000; 4.54	*Cracticus nigrogularis picatus*	Tony Baylis
Woods near Cumberdeen Dam, Pilliga Forest, New South Wales	22/10/2001; 4.30	*Cracticus nigrogularis nigrogularis*	Dr Jenny Beasley
Stuart/Ross Hwys, S of Alice Springs, Northern Territory	08/10/2006; 4.15	*Cracticus nigrogularis picatus*	Dr Hollis Taylor
Wordsworth Road off Flinders Hwy, Townsville to Charters Towers, north Queensland	28/09/2007; 4.10	*Cracticus nigrogularis nigrogularis*	Dr Hollis Taylor
King's Creek Station, Northern Territory	3/09/2000; 5.15	*Cracticus nigrogularis picatus*	Dr David Lumsdaine
Palm Valley, Finke Gorge NP, Northern Territory	3/09/1993; 5.23	*Cracticus nigrogularis picatus*	Vicki Powys
Indooroopillly, Queensland	23/10/1995; pre-dawn	*Cracticus nigrogularis nigrogularis*	Dr Gayle Johnson
Carey Street, Bardon, Queensland	01/09/1998; pre-dawn	*Cracticus nigrogularis nigrogularis*	Dr Gayle Johnson
Broome, Western Australia	11/10/1992; pre-dawn	*Cracticus nigrogularis picatus*	Dr Peter Fullagar
9 Magpie Lane, Gowrie Junction, Queensland	6/09/1992; pre-dawn	*Cracticus nigrogularis nigrogularis*	Gloria Glass
Uluru National Park, Northern Territory	28/09/2006; 4.38	*Cracticus nigrogularis picatus*	Dr Hollis Taylor
9 Magpie Lane, Gowrie Junction, Queensland	29/09/1998–01/10/98; pre-dawn	*Cracticus nigrogularis nigrogularis*	Gloria Glass
Nelly Bay, Magnetic Island, north Queensland	15/06/2005; 12.24	*Cracticus nigrogularis nigrogularis*	Dr Hollis Taylor
Wogarno Station, Western Australia	21/11/2008; 5.15	*Cracticus nigrogularis picatus*	Dr Hollis Taylor
Ross River Resort Campground, E of Alice Springs, Northern Territory	11/09/2007; 4.35	*Cracticus nigrogularis picatus*	Dr Hollis Taylor
Nelly Bay, Magnetic Island, north Queensland	14/06/2005; 15.03	*Cracticus nigrogularis nigrogularis*	Dr Hollis Taylor
9 Magpie Lane, Gowrie Junction, Queensland	06/10/2002; pre-dawn	*Cracticus nigrogularis nigrogularis*	Gloria Glass

### 5.2. Sound data pre-processing

Each recording was pre-processed using Goldwave®: we filtered the data using a bandpass filter of 0.5–3 kHz (the pied butcherbird song frequency range), and then used the Goldwave noise reduction function to further reduce environmental background noise, by sampling intervals of stationary background noise and subtracting the noise power spectrum from the signal. Pied butcherbird phrases were then identified by the 2–3 s pauses that separate them (figure 2*a*). Song phrases were saved into separate files, indexed by phrase temporal order, and were then analysed using Matlab 8, including signal processing and statistics toolboxes.

### 5.3. Unsorted and sorted raster plots of entire singing performances

In order to inspect and quantify the entire singing performance of each bird, we simplified the sonograms of phrases into a time course (vector) of pitch values, and presented it as a false-colour bar (figure 2*b*). We then stacked those bars as rows on top of one another to produce raster plots of entire singing performances (figure 2*c*), where each line represents a phrase, aligned by the onset of the first syllable. The unsorted raster plots are stacked according to phrases in performance order (figure 2*b*,*c*,*e*). The sorted raster plots (figures 2*d*,*f* and 3*d*,*g*) present phrases according to their type, which we calculated semi-automatically as described in the next section. We developed a Graphic User Interface (GUI) in Matlab (http://www.ethankeyboards.com/locateflow/locateflow/online-methods/), which allows us to point-and-click, to rearrange phrases vertically and to play phrases (in real speed or slow motion) by clicking on each phrase type. We used these GUIs as descriptive models that could facilitate perceiving those performances holistically, combining visual and auditory perception to detect similarities and differences across all the phrase types produced by each bird.

### 5.4. Automatic classification of phrase types

Different approaches were used to estimate the song repertoire in pied butcherbirds. One approach is to identify each phrase type as a stereotyped ‘core’ consisting of three or more notes, with variable beginnings and endings. In a sample of 27 birds, this approach gave repertoires varying between 1 and 10 phrase types and an average of five (H Taylor & C Scharff 2015, unpublished data). The weakness of this approach is the difficulty of deciding how much variation should be allowed within a phrase type. An alternative approach is of identifying a phrase as a unique occurrence of a string of notes that is delivered multiple times in exactly the same way. This approach gives a repertoire ranging between 3 and 43 phrase types, with an average of 21 in the analysed cohort of 27 birds mentioned above. Here, we used an automated procedure for identifying both unique occurrences, and core elements, which we call ‘shared motifs’. Owing to the complexity of the pied butcherbird song phrases, simple measures such as duration and mean pitch do not distinguish between them. By contrast, the temporal structure (note onset time) was unique to each phrase type. Therefore, based solely on the onset times of syllables, we used a hierarchical clustering technique called spike sorting to automate the determination of phrase types. We extracted the onset time of syllables within each phrase to transform each phrase into binary data with ones representing syllable onsets (a point process), and used a spike-sorting algorithm to automatically detect and sort phrases types using the following steps:

— The phrase amplitude was normalized using the Matlab wavesc function.
— We automatically identified syllable onsets by smoothing data, computing the amplitude derivative and identifying local maxima. Local maxima above an empirically determined threshold were marked as syllable onsets.
— We converted the amplitude time course into a binary signal (sampled at 1000 Hz) where syllable onsets were labelled as 1, and all other time points as zero. We then treated those data as a point process for spike sorting, such that the first syllable onset was assigned as the beginning of the phrase, to align the vectors.
— We used the Spike Train Communities Toolbox for Matlab [39] to automatically classify the song phrases into types. We used the spike-sorting code as is, without modification, using the default parameters. The spike-sorting algorithm then grouped phrases into categories (types; figure 2*f*). We then reconstructed the false-colour map of the sorted phrases as shown in figure 2*d*. Using a GUI described above, we inspected the clusters, and when needed, we further sorted them until all colour bars were grouped. This last stage, which is semi-automatic, was used to identify virtually identical phrase types. Therefore, our criteria for defining phrase types is highly conservative, with each ‘variant’ in order of syllables, no matter how minor, classified as a distinct phrase type.

### 5.5. Classification of motifs

The sorted false-colour maps of song phrases facilitated visual identification of shared motifs, which were re-used in multiple phrase types. We successively identified motifs manually until all the visually similar motifs had been grouped together. We used our visual sorting method to identify candidate motifs, we then listened to them, and inspected all their acoustic features side-by-side to confirm that they are virtually identical across phrases in all features. Here too, we used a conservative approach and considered only cases where we failed to detect any deviation in any acoustic feature.

A visual map was constructed using the methodology seen in figure 3*d*. Motifs were defined as syllables or syllable sequences that were found in multiple contexts (figure 3*a*,*c*,*f*) in different phrases. Repeated syllables, as in the case of B in ABB, were treated as one motif (BB is a motif). However, if extended repetition was also found, as in ABBB, then the more basic unit (here B can be added to BB) was identified as a motif (B is the motif).

We chose this top-down approach of motif classification because visual identification of syllables as a first step had its own biases. Further, automated syllable classification was biased by thresholding, and errors due to noise could leave syllables misclassified. Classification of motifs was accomplished blind to phrase sequence patterning.

Each phrase could now be represented as a sequence of motif names (figure 3*c*). The phrases, represented as a sequence of motifs, were entered into a spreadsheet and arranged according to the original singing order (one phrase per row, one motif per cell). The spreadsheet data was imported into Matlab for analysis as a ‘motif matrix’.

### 5.6. Analysis of motif distribution

To measure how consistently a motif was re-used within a bird's sequence of phrases, we created a summary statistic to quantify the regularity in the distribution of interphrase intervals (IPIs, figure 4*e*) for all motifs across each bird's performance. We used CV as a measure of uniformity in the distribution of IPIs from one appearance of a motif to its next appearance. We calculated IPIs for each motif and divided each interval of that set by the mean of the IPIs for that motif and called this the ‘normalized set of IPIs’ value for each motif.

5.1 $$\mathrm{IPI}s_{\mathrm{normalized}}=\frac{\left(\mathrm{IPIs}\right)}{\mathrm{mean}(\mathrm{IPIs})}.$$

Then we calculated a CV value (s.d./mean) across the set of all normalized IPIs to gain a general sense of the level of variability within a bird's performance. This summary statistic will hereafter be simply referred to as CV*.

5.2 $$\mathrm{IPI}s^{*}=(\mathrm{IPI}s_{1},\mathrm{IPI}s_{2},\mathrm{IPI}s_{3}\ldots )$$

and

5.3 $${\mathrm{CV}}^{*}=\frac{\sigma (\mathrm{IPI}s^{*})}{\mu (\mathrm{IPI}s^{*})}.$$

### 5.7. Statistical models

The duration of recording, which varied from 5 min to 1 h across birds, might have influenced the assessment of repertoire and derived measures. To avoid such artefacts, we used only scale invariant measures in our statistical models. We used three statistical models in order to assess the temporal structure of phrases and motifs: ‘Shuffled’, ‘Bigram Markov’ and ‘Permuted Markov’.

#### 5.7.1. Shuffled model

For each bird, the rows of the motif matrix corresponding to their natural singing behaviour were randomly shuffled by assigning each phrase with a pseudo-random number, and then sorting phrases order according to those random numbers.

#### 5.7.2. Bigram Markov model

For each individual recording sampled from a bird (we will refer to it as ‘bird’ hereafter), transition probabilities between phrase types were calculated from the bird's natural singing order, Bigrams_Matrix_bird_. Using this matrix, we generated synthetic strings as long as the birds performance: after selecting a phrase at random to start the sequence, the remaining phrases were chosen based on the transition probabilities from the original performance until the same number of phrases as the original performance had been chosen. CV* was then calculated (equation (5.3)). We repeated this procedure 100 times, and computed the average CV* value across all the bigram Markov sequences. This value was chosen to represent the ${\mathrm{CV}}_{\left[\mathrm{synth}\text{-}\mathrm{bird}\right]}^{*}$ of a ‘bigram Markov’ model.

#### 5.7.3. Permuted Markov model

We generated permutations of the Markov model by using the transition probabilities of the original model but then permuting which phrases were assigned to which transitions (figure 5*a*). In total, 100 permuted models were used to represent the space of possibilities for alternative Markov models that still maintained the basic structure of transition probabilities. For each permutation, 100 models were generated and an average ${\mathrm{CV}}_{\left[\mathrm{synth}\text{-}\mathrm{permuted}\right]}^{*}$ value was calculated for each sequence generated by a new permutation (figure 5*b*).

### 5.8. Statistical analysis

To compare the CV* achieved by a model with the CV* achieved by original singing behaviour, we used a paired *t*-test. Note that our CV* measures are mean values, and therefore a parametric *t*-test is appropriate. Each bird's original CV* value was subtracted from the CV* value generated by the model from that bird's data to provide a set of difference scores. To estimate a correlation between ranks and CV*s of Markov models and ranks of CV* among permuted models (figure 5*d*), we used the bivariate correlation function in SPSS.

### 5.9. Complexity measures

To capture the complexity of each bird's bigram Markov model, we use the number of non-zero elements in the associated Markov transition matrix. This corresponds to the concept of Kolmogorov complexity, which is defined as the shortest instruction set, or ‘programme’, that can generate a sequence. In this case, the transition matrix can generate a sequence comparable to the one performed by the bird and the matrix elements are all that are required to ‘execute the programme’. The length of the programme is given by the sequence describing the location and value of each non-zero element in the matrix.

## Supplementary Material

S1. Cohort 1, sorted and unsorted raster plots.

## Supplementary Material

S2. Cohort 2, sorted and unsorted raster plots.

## Supplementary Material

ST1: analysis of variance between cohort 1 (9 birds) and cohort 2 (8 birds):

## Data accessibility

Field recordings of butcherbirds we analysed can be accessed in the Dryad Digital Repository http://dx.doi.org/10.5061/dryad.9nv3b [62]. Online Methods tutorials are available at http://www.ethankeyboards.com/locateflow/locateflow/online-methods.
