# Supplementary figures and images for: Temporal regularity increases with repertoire complexity in the Australian pied butcherbird's song
Source: R Soc Open Sci. 2016 Sep 14;3(9):160357. doi: 10.1098/rsos.160357 (PMC5043318; doi:10.1098/rsos.160357)

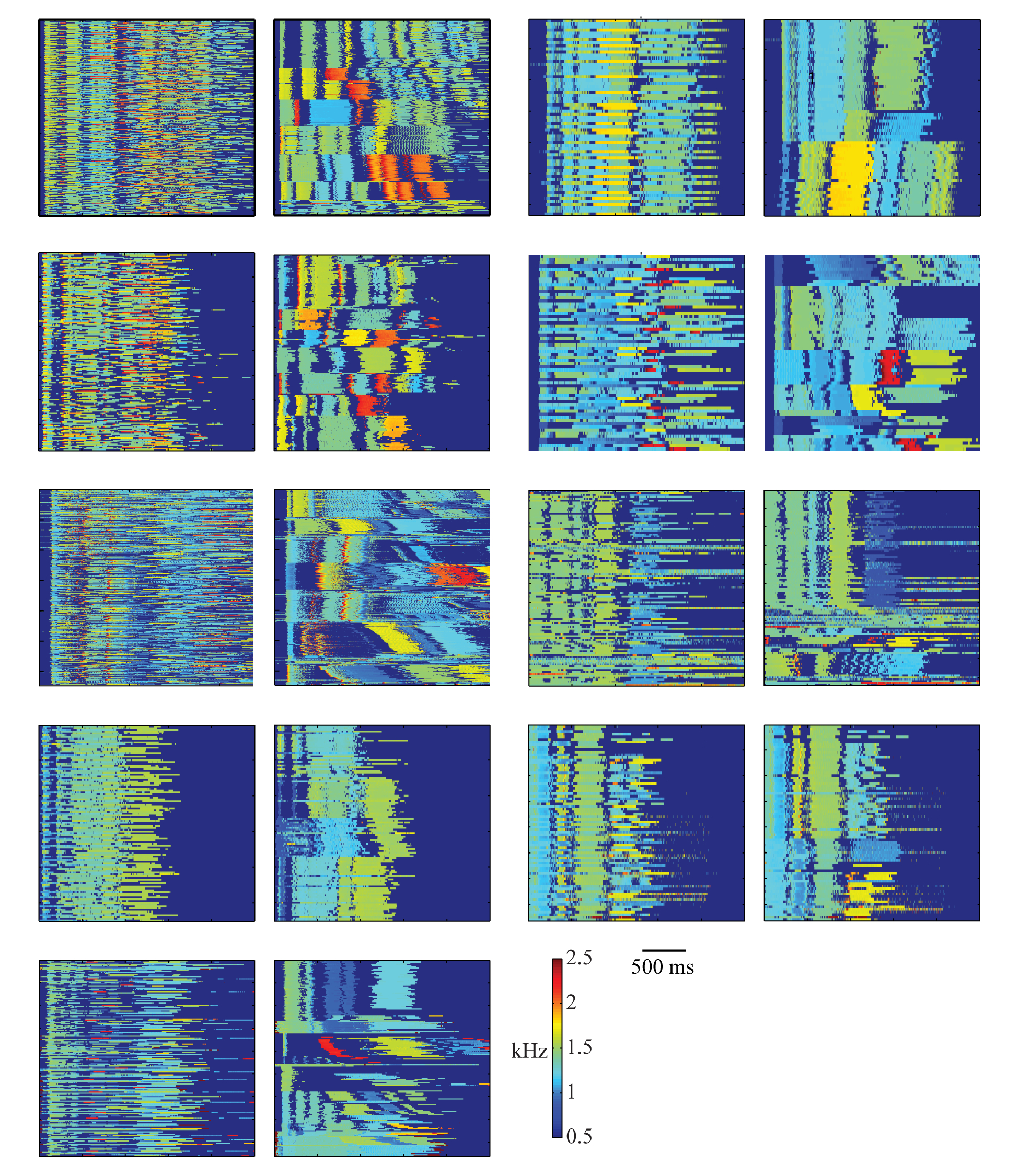

Supplement: S1. Cohort 1, sorted and unsorted raster plots. [file rsos160357supp1.jpg]

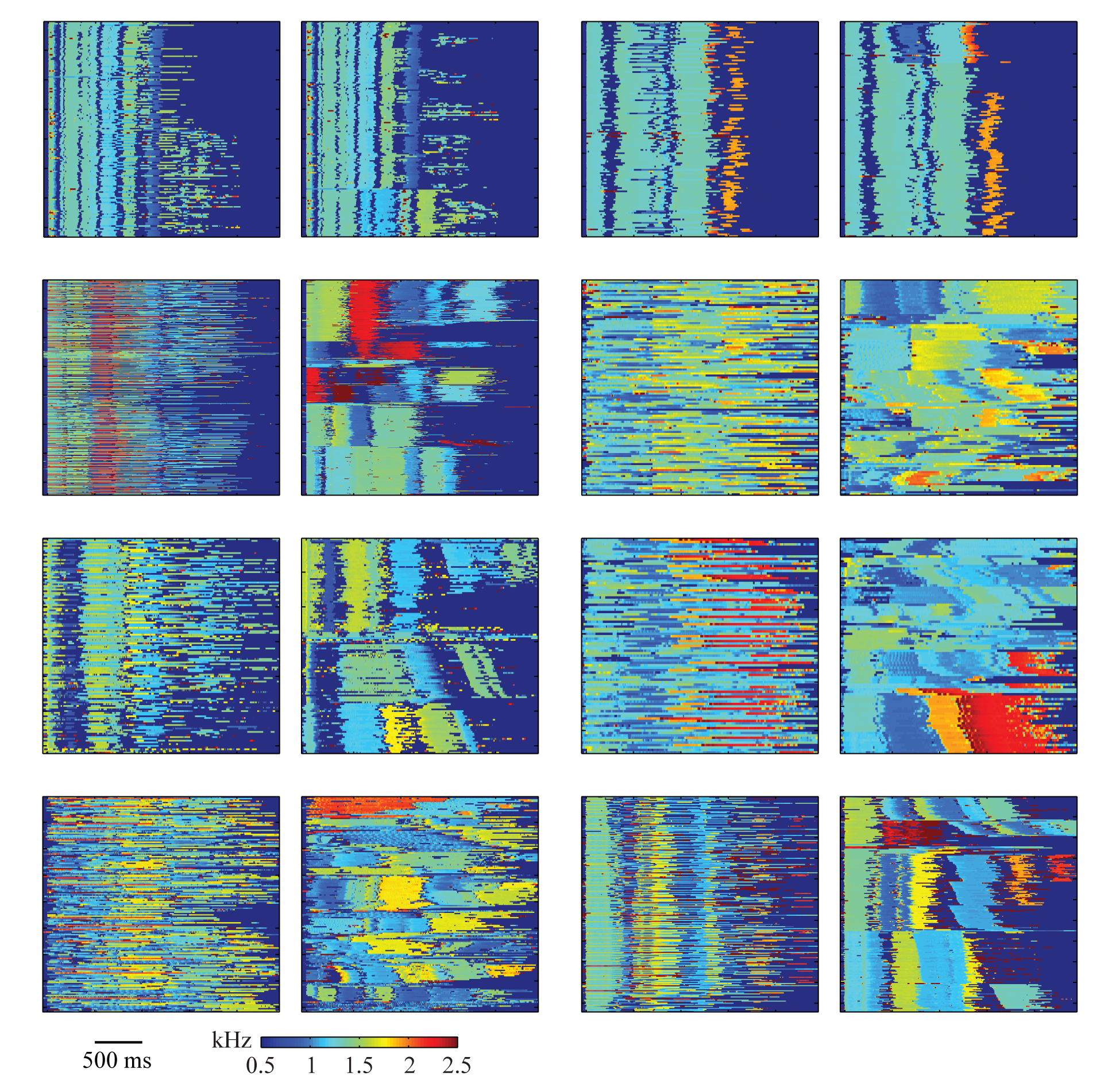

Supplement: S2. Cohort 2, sorted and unsorted raster plots. [file rsos160357supp2.jpg]
