# Supplementary material for: Temporal regularity increases with repertoire complexity in the Australian pied butcherbird's song
Source: R Soc Open Sci. 2016 Sep 14;3(9):160357. doi: 10.1098/rsos.160357 (PMC5043318; doi:10.1098/rsos.160357)
Supplement: ST1: analysis of variance between cohort 1 (9 birds) and cohort 2 (8 birds): [file rsos160357supp3.docx]

| Multivariate Tests^a^ | | | | | | | |
| --- | --- | --- | --- | --- | --- | --- | --- |
| Effect | | Value | F | Hypothesis df | Error df | Sig. | Partial Eta Squared |
| motif / phrase | Wilks' Lambda | 0.546 | 12.491b | 1 | 15 | 0.003 | 0.454 |
| (motif / phrase) * cohort | Wilks' Lambda | 0.973 | .418b | 1 | 15 | 0.528 | 0.027 |
| shuffle / bird | Wilks' Lambda | 0.397 | 22.822b | 1 | 15 | 0 | 0.603 |
| (shuffle / bird) * cohorts | Wilks' Lambda | 0.817 | 3.365b | 1 | 15 | 0.086 | 0.183 |
| (motif / phrase) * (shuffle / bird) | Wilks' Lambda | 0.708 | 6.188b | 1 | 15 | 0.025 | 0.292 |
| (motif / phrase) * (shuffle / bird) * cohort | Wilks' Lambda | 0.934 | 1.057b | 1 | 15 | 0.32 | 0.066 |
| a. Design: Intercept + cohorts  Within Subjects Design: motif_phrase + shuffle_bird + motif_phrase * shuffle_bird | | | | | | | |

| Tests of Between-Subjects Effects | | | | | | |
| --- | --- | --- | --- | --- | --- | --- |
| Measure: MEASURE_1  Transformed Variable: Average | | | | | | |
| Source | Type III Sum of Squares | df | Mean Square | F | Sig. | Partial Eta Squared |
| Intercept | 30.915 | 1 | 30.915 | 650.919 | .000 | .977 |
